# Suicidality and Hostility following Involuntary Hospital Treatment

**DOI:** 10.1371/journal.pone.0154458

**Published:** 2016-05-12

**Authors:** Domenico Giacco, Stefan Priebe

**Affiliations:** Unit for Social and Community Psychiatry, WHO Collaborative Centre for Mental Health Service Development, Queen Mary University of London, London, United Kingdom; Yokohama City University, JAPAN

## Abstract

**Background:**

Psychiatric patients showing risk to themselves or others can be involuntarily hospitalised. No data is available on whether following hospitalisation there is a reduction in psychopathological indicators of risk such as suicidality and hostility. This study aimed to assess changes in suicidality and hostility levels following involuntary admission and their patient-level predictors.

**Methods:**

A pooled analysis of studies on involuntary treatment, including 11 countries and 2790 patients was carried out. Suicidality and hostility were measured by the Brief Psychiatric Rating Scale.

**Results:**

2790 patients were included; 2129 followed-up after one month and 1864 after three months. 387 (13.9%) patients showed at least moderate suicidality when involuntarily admitted, 107 (5.0%) after one month and 97 (5.2%) after three months. Moderate or higher hostility was found in 1287 (46.1%) patients after admission, 307 (14.5%) after one month, and 172 (9.2%) after three months. Twenty-three (1.2%) patients showed suicidality, and 53 (2.8%) patients hostility at all time-points. Predictors of suicidality three months after admission were: suicidality at baseline, not having a diagnosis of psychotic disorder and being unemployed. Predictors of hostility were: hostility at baseline, not having a psychotic disorder, living alone, and having been hospitalized previously.

**Conclusions:**

After involuntary hospital admission, the number of patients with significant levels of suicidality and hostility decreases substantially over time, and very few patients show consistently moderate or higher levels of these symptoms. In patients with psychotic disorders these symptoms are more likely to improve. Social factors such as unemployment and isolation could hamper suicidality and hostility reduction and may be targeted in interventions to reduce risk in involuntarily admitted patients.

## Introduction

Across the world, severely mentally ill patients can be involuntarily admitted to hospital during the acute episodes of their illness, and such admissions are widely practiced [1–6]. Systematic research is required to investigate whether this practice is justified or not. Randomized controlled trials testing the effectiveness of involuntary hospital admissions as compared to non-coercive forms of treatment may be desirable, but remain very difficult to conduct for various ethical and practical reasons. Thus, best evidence is obtained based on observational studies, following up cohorts of patients exposed to involuntary treatment.

Existing observational studies have suggested only limited improvements of general symptoms and minimal, if any, social gains following involuntary admission [4,7]. It has been argued, however, that the main aim of involuntary hospital treatment of patients is not the improvement of general symptoms or of the social situation, but the reduction of risk [3,6]. To date, no large scale studies have been published showing to what extent the risk for suicide and aggression really decreases after involuntary hospital treatment.

We analyzed data from the two largest observational studies on outcomes of involuntary hospital treatment available to date, focusing on psychopathological indicators of risk, i.e. suicidality and hostility. The studies used an identical methodology for assessing both baseline characteristics and outcomes of patients. Although various findings of these studies have been published, so far no specific analysis of risk indicators has been conducted [2,5,7].

Suicidality and hostility were assessed by researchers who were not involved in treatment. The advantages of these measures are that they are independent of treating clinicians, who may have biased views on the patients’ actual risk indicators; can be assessed consistently across different countries and settings; and reflect clinical symptoms that may be targeted in treatment.

The two studies have very similar designs [2,5], which enabled us to conduct a pooled analysis and test associations of patient characteristics with suicidality and hostility outcomes. We considered socio-demographic and clinical characteristics of patients that have been found to be linked with risk in the literature (age, gender, employment, living situation, past hospitalizations, diagnosis and global functioning).

The specific research questions were:

How many patients show moderate or higher levels of suicidality and hostility when involuntarily admitted, and how many patients have such levels one month and three months later?

How many patients show such levels consistently, i.e. when involuntarily admitted, after one month and after three months?

What patient characteristics predict suicidality and hostility three months after involuntary hospital admission?

## Materials and Methods

### Design and selection of participants

We carried out a ‘‘pooled analysis”, i.e. individual patient data within the studies were pooled in a larger dataset and analysed. This approach enabled a precise estimate of effects of influential and confounding factors, and takes into account the heterogeneity of countries [8].

Data from two observational prospective studies [2,5] were included in the analysis.

The first study was the “European Evaluation of Coercion in Psychiatry and Harmonisation of Best Clinical Practice (EUNOMIA)”. It assessed the outcomes of involuntarily admitted patients in 11 European countries (Germany, Bulgaria, Czech Republic, Greece, Italy, Lithuania, Poland, Slovak Republic, Spain, Sweden and the United Kingdom). In each country between one and five hospitals were included. Data was collected between July 2003 and October 2005.

The second study was the “Outcomes of involuntary hospital admission in England (InvolvE)”. This was conducted in 22 hospitals within eight Trusts (provider organisations) of the National Health Service in England. Data was collected between July 2003 and July 2005.

In both studies the inclusion criteria were: involuntarily admitted patients; 18 to 65 years of age; sufficient command of the national language, and capacity to provide written informed consent. Patients were excluded if they had a primary diagnosis of dementia; were admitted due to intoxication, or were transferred from a different hospital [2,5].

The detailed methods and general findings of the two studies have been described in previous publications [2,5].

### Ethics statement

The EUNOMIA study was approved by the relevant Research Ethics committees in each country [2]:

- Research Ethics Committee, Medical University Sofia, Sofia, Bulgaria
- The Ethics Committee of the General Teaching Hospital, Prague, Czech Republic
- Ethics committee at the Faculty of Medicine at Dresden University of Technology, Dresden, Germany
- Scientific Board of the Psychiatric Hospital of Thessaloniki, Thessaloniki, Greece
- Ethical Committee of the Second University of Naples, Naples, Italy
- Lithuanian Bioethical Committee, Vilnius, Lithuania
- Commission of Bioethics at Wroclaw Medical University, Wroclaw, Poland
- Ethical Committee of the Michalovce Psychiatric Hospital, Michalovce, Slovak Republic
- Ethical Committee (Comité Ético) of University Hospital of San Cecilio. Granada, Spain
- Research Ethics Committee of Örebro University Hospital, Örebro, Sweden
- East London and The City Research Ethics Committee, London, UK

The InvolvE study received ethical approval from the UK Multi-Centre Research Ethics Committee (ref. MREC/03/0/96) [5].

For both studies, written informed consent was obtained from all participants. This procedure was approved by Ethics Committees in each country.

### Procedures and Measures

In both studies, patients who consented to take part in the study were first interviewed within a week after involuntary admission (i.e. at the study baseline) and then followed-up after one and three months.

Suicidality and hostility were measured by the item 4 (suicidality) and item 6 (hostility) on the Brief Psychiatric Rating Scale (BPRS) [9]. These items are scored on a scale from 1 (not present) to 7 (extremely severe) and cover the week before the assessment, including the admission period.

Researchers in both studies were trained in the assessment, achieving a good inter-rater reliability for the BPRS score in the EUNOMIA study (Cohen’s kappa = 0.78) [2] and a very high inter-rater reliability in the InvolvE study (Cohen’s kappa = 0.90) [5].

Suicidality and hostility scores were dichotomised as scores of <4 versus those of ≥4. The latter reflects a moderate or more severe level of the given symptom [9]. For suicidality, a score of 4 indicates that “frequent suicidal thoughts without intent or plan are reported by the patient”, whilst a 3 indicates “occasional suicidal thoughts without intent or specific plan OR he/she reports they would be better off dead”. With respect to hostility, a 4 is rated when a patient “was overtly angry on several occasions OR yelled at others excessively” and a 3 is rated when the patient is “argumentative or sarcastic”.

Data on socio-demographic characteristics, clinical history and diagnosis according to ICD-10 categories [10] were gathered from medical records. Diagnoses were collapsed into 3 groups: schizophrenia or other psychotic disorders, i.e. categories F20-29; affective disorder, i.e. categories F30-39; and other disorders. Researchers also rated patients’ Global Assessment of Functioning (GAF) scoring 0 to 100, with 100 indicating the highest functioning [11]. The numbers and percentages of people who were in hospital after three months from admission (either because the index admission was still ongoing or they were re-admitted to the same or a different hospital) were calculated. We tested the associations between being in hospital and showing a significant suicidality or hostility through univariate logistic regression models controlled for whether patients were significantly suicidal or hostile at admission.

### Statistical analysis

Descriptive statistics on baseline socio-demographic and clinical characteristics of patients were calculated for the total sample. The dichotomised scores on suicidality and hostility scores were calculated for each time point.

Basic socio-demographic and clinical characteristics were tested as potential predictors of suicidality and hostility three months after admission: gender, age, employment, living situation, past hospitalisations, diagnosis (collapsed into three groups), and global functioning (GAF score). Univariable and multivariable logistic regression analyses were carried out to test associations. We used logistic regression models and dichotomous outcome variables as the distribution of values of both suicidality and hostility at all time points was highly skewed to the left, which would violate assumptions for a linear regression model. All variables that were found significant at p = .10 in univariable analyses were subsequently considered in multivariable logistic regression model analyses, adjusted for country effect. Country effects were controlled for by fitting dummy variables for each individual country. Logistic regression models diagnostics, i.e. the Hosmer and Lemeshow Goodness of fit and c-index were computed for the two multivariable models. Adjusted percentages were calculated for variables which showed associations with either suicidality or hostility.

## Results

### Sample characteristics

Out of the 6003 eligible patients in the two studies, 2790 (46.5%) gave informed consent to participate and were interviewed within a week following admission (baseline); 2129 were followed up after one month and 1864 after three months. Reasons for not participating in the baseline or follow-up interviews have been specified elsewhere [2,5].

In the included sample at baseline (n = 2790), 387 (14.0%) patients showed moderate or higher levels of suicidality and 1287 (46.1%) of hostility; 159 (5.7%) had both symptom. Overall, slightly more than half of the patients (54.1%) had either suicidality or hostility at baseline; at the three month follow up 552 (19.8%) were in hospital.

The socio-demographic and clinical characteristics of the total sample and of patients with at least moderate levels of suicidality and hostility are reported in Table 1.

**Table 1:** Socio-demographic and clinical characteristics of the total sample and of patients with moderate or high levels of suicidality and hostility at baseline.

Variables	Total sample (n = 2770)	Patients with moderate or high levels of suicidality (n = 387)	Patients with moderate or high levels of hostility (n = 1287)
Gender, male, n (%)	1529 (55.2)	205 (53.0)	757 (58.8)
Age, years, mean (s.d.)	38.9 (11.5)	37.7 (11.5)	38.0 (10.9)
Employment, yes, n (%)	598 (21.1)	72 (18.6)	260 (20.2)
Living alone, yes, n (%)	1864 (65.7)	246 (63.6)	895 (69.5)
Previous hospital admission, yes, n (%)	1985 (71.2)	280 (72.4)	942 (73.2)
Diagnosis			
Psychotic disorders, n (%)	1618 (59.2)	138 (35.7)	769 (59.8)
Affective disorders, n (%)	492 (18.0)	103 (26.6)	239 (18.6)
Other disorders, n (%)	660 (23.8)	146 (37.7)	279 (21.7)
GAF score, mean (s.d.)	32.2 (14.2)	29.2 (14.3)	29.3 (13.1)
Length of hospitalization, days, mean (s.d.)	54.9 (70.1)	51.7 (68.9)	46.1 (59.8)

### Suicidality

One month after admission, 107 patients had moderate or higher levels of suicidality (5.0% of assessed patients), and 97 patients had such symptoms after three months (5.2%). Twenty-three patients were consistently rated as suicidal (0.8% of those followed up throughout the study). After three months 33 (34%) of the people who showed significant suicidality were in hospital. People who consistently showed significant suicidality at admission and three months were more likely to be in hospital at three months (OR = 1.659, p = .031).

There was some variation between countries. The percentage of patients with suicidality after three months ranged from 0% (Lithuania) to 16.7% (Sweden).

The percentage of patients with consistent suicidality ranged from 0% (in Bulgaria, Czech Republic, Greece, Italy, Lithuania, Poland, Slovakia) to 7.4% (Sweden).

Numbers and percentages of patients with moderate or higher levels of suicidality for each country and time point are shown in Table 2.

**Table 2:** Patients with moderate or high levels of suicidality (M/HS) in the participating countries.

		When involuntarily admitted	One month follow up	Three-month follow-up	Consistently M/HS
Bulgaria	*Assessed*, *N*	309	297	289	289
	*M/HS*, *N (%)*	19 (6.1)	5 (1.7)	1 (0.3)	0 (0)
Czech Republic	*Assessed*, *N*	201	165	144	144
	*M/HS*, *N (%)*	37 (18.4)	6 (3.6)	1 (0.7)	0 (0)
Germany	*Assessed*, *N*	145	120	106	106
	*M/HS*, *N (%)*	35 (24.1)	9 (6.2)	4 (3.8)	2 (1.9)
Greece	*Assessed*, *N*	205	164	141	141
	*M/HS*, *N (%)*	15 (6.8)	12 (5.4)	7 (3.2)	0 (0)
Italy	*Assessed*, *N*	125	114	92	92
	*M/HS*, *N (%)*	11 (8.8)	1 (0.9)	1 (1.1)	0 (0)
Lithuania	*Assessed*, *N*	85	66	48	48
	*M/HS*, *N (%)*	10 (11.8)	0 (0)	0 (0)	0 (0)
Poland	*Assessed*, *N*	152	136	134	134
	*M/HS*, *N (%)*	12 (7.9)	0 (0)	4 (3.0)	0 (0)
Slovak Republic	*Assessed*, *N*	296	221	162	162
	*M/HS*, *N (%)*	29 (9.8)	3 (1.4)	2 (1.2)	0 (0)
Spain	*Assessed*, *N*	418	258	236	236
	*M/HS*, *N (%)*	65 (15.6)	14 (5.4)	26 (11.0)	6 (2.5)
Sweden	*Assessed*, *N*	92	59	54	54
	*M/HS*, *N (%)*	16 (17.4)	7 (11.9)	9 (16.7)	4 (7.4)
United Kingdom	*Assessed*, *N*	760	529	457	457
	*M/HS*, *N (%)*	138 (18.2)	50 (9.5)	42 (9.2)	11 (2.4)

### Hostility

One month after admission, 307 of the assessed patients had moderate or higher levels of hostility (14.5%), and 172 (9.2%) after three months. The percentage of patients with such hostility levels at three months varied from 0% (Lithuania) to 17.1% (Spain). In all countries, the percentage of patients with hostility decreased substantially between baseline and three months.

Fifty-three patients showed hostility consistently (2.1% of those followed up throughout the study). Continuous hostility was observed in a percentage of patients ranging from 0% (in Lithuania, Poland and Slovakia) to 4.8% (Spain).

After three months 72 (41.9%) of the people who showed significant hostility were in hospital. People who consistently showed significant hostility at admission and three months were more likely to be in hospital at three months (OR = 2.208; p < .001).

Numbers and percentages of patients with moderate or high levels of hostility are reported per country and time point in Table 3.

**Table 3:** Patients with moderate or high levels of hostility (M/HH) in the participating countries.

		When involuntarily admitted	One month follow up	Three-month follow-up	Consistently M/HH
Bulgaria	*Assessed*, *N*	309	297	289	289
	*M/HH*, *N (%)*	200 (64.7)	75 (25.3)	21 (7.3)	14 (4.8)
Czech Republic	*Assessed*, *N*	201	165	144	144
	*M/HH*, *N (%)*	65 (32.3)	9 (5.5)	10 (6.9)	3 (2.1)
Germany	*Assessed*, *N*	145	120	105	105
	*M/HH*, *N (%)*	82 (56.6)	20 (16.7)	5 (4.8)	3 (2.9)
Greece	*Assessed*, *N*	208	161	139	139
	*M/HH*, *N (%)*	162 (73.0)	33 (20.5)	20 (14.4)	5 (3.6)
Italy	*Assessed*, *N*	127	115	97	97
	*M/HH*, *N (%)*	83 (65.4)	21 (16.3)	12 (12.4)	4 (4.1)
Lithuania	*Assessed*, *N*	85	66	48	48
	*M/HH*, *N (%)*	23 (27.1)	4 (6.1)	0 (0)	0 (0)
Poland	*Assessed*, *N*	152	136	135	135
	*M/HH*, *N (%)*	31 (20.4)	6 (4.4)	4 (3.0)	0 (0)
Slovak Republic	*Assessed*, *N*	296	221	162	162
	*M/HH*, *N (%)*	80 (27.0)	4 (1.8)	4 (2.5)	0 (0)
Spain	*Assessed*, *N*	418	253	234	234
	*M/HH*, *N (%)*	164 (39.2)	32 (12.6)	40 (17.1)	8 (2.3)
Sweden	*Assessed*, *N*	95	58	54	54
	*M/HH*, *N (%)*	34 (35.8)	7 (12.1)	6 (11.1)	2 (3.7)
United Kingdom	*Assessed*, *N*	754	529	457	457
	*M/HH*, *N (%)*	363 (48.1)	96 (18.1)	50 (10.9)	14 (3.1)

### Patient characteristics predicting risk three months after admission

#### Suicidality

Univariable and multivariable models testing associations of socio-demographic and clinical characteristics considered with suicidality levels at three-month follow up are reported in Table 4.

**Table 4:** Predictors of at least moderate suicidality three months after involuntary hospital admission (n = 1864).

Covariates	Univariable model				Multivariable model^a^^,^^b^			
	*OR*^c^	95% CI^d^		*P*-value	*OR*^c^	95% CI^d^		*P*-value
		Lower bound	Upper bound			Lower bound	Upper bound	
Gender								
Male vs. Female	1.227	.815	1.847	.327				
Age	.985	.967	1.003	.100	.988	.968	1.008	.251
Employed vs. Unemployed	.447	.230	.870	.018	.415	.206	.837	.014
Living alone; Yes vs. No	1.180	.771	1.805	.446				
Previous hospital admission, Yes vs. No	1.177	.728	1.905	.506				
Diagnosis								
Schizophrenia and related disorders (F20-29) vs. others	.287	.185	.445	< .001	.338	.196	.582	< .001
Affective disorders (F30-39) vs. others	1.528	.954	2.446	.077	.549	.304	.991	.047
Global Assessment of Functioning score	.993	.979	1.008	.353				
At least moderate suicidality at baseline, Yes vs. No	7.926	5.156	12.186	< .001	5.788	3.622	9.248	< .001
At least moderate hostility at baseline, Yes vs. No	.935	.619	1.412	.749				

^a^ Controlled for countries’effects

^b^ The Hosmer and Lemeshow Goodness of Fit. Test statistics were: Chi-square = 5.439; df = 8, p = .71. The C-index was: 0.846; 95% CI = .808-.884; Standard Error = .019, Asymptotic sig. < .001. The values of both tests indicated good fit of the multivariable model.

^c^ OR = Odds ratio

^d^ CI = Confidence Interval

In the univariable models, being unemployed and having at least moderate suicidality at baseline were associated with higher likelihood of being suicidal at three months, whilst having a psychotic disorder (F20-29) was associated with a reduced likelihood of suicidality at three months. All these associations held true in the multivariable model, adjusted for countries’ effect.

At three months, 2.8% (Adjusted Percentage based on multivariable logistic regression model, AP = 3·1%) (N = 31) of patients with psychotic disorders were rated as suicidal as compared with 9.0% (AP = 7.8%) (N = 66) of patients with non-psychotic disorders. With respect to employment, 5.8% (AP = 2.8%) (N = 87) of unemployed (AP = 5.9%) and 2.7% (N = 10) of employed patients were rated as suicidal.

#### Hostility

Univariable and multivariable models testing associations of socio-demographic and clinical characteristics considered with hostility levels at three-months are reported in Table 5.

**Table 5:** Predictors of at least moderate hostility three months after involuntary hospital admission (n = 1864).

Covariates	Univariable model				Multivariable model^a^^,^^b^			
	*OR*^c^	95% CI^d^		*P*-value	*OR*^c^	95% CI^d^		*P*-value
		Lower bound	Upper bound			Lower bound	Upper bound	
Gender								
Male vs. Female	.719	.521	.993	.045	.856	.594	1.233	.404
Age	.992	.978	1.006	.237				
Employed vs. Unemployed	.596	.378	.939	.026	.660	.407	1.069	.091
Living alone, Yes vs. No	2.053	1.438	2.930	< .001	1.528	1.031	2.262	.034
Previous hospital stay Yes vs. No	1.725	1.152	2.584	.008	1.739	1.131	2.676	.012
Diagnosis								
Schizophrenia and related disorders (F20-29) vs. others	.676	.493	.925	.015	.459	.308	.685	< .001
Affective disorders (F30-39) vs. others	.856	.564	1.299	.466	.436	.261	.730	.002
Global Assessment of Functioning score	.993	.982	1.005	.244				
At least moderate hostility at baseline, Yes vs. No	2.256	1.618	3.145	< .001	2.010	1.395	2.896	< .001
At least moderate suicidality at baseline, Yes vs. No	1.473	.973	2.229	.067	1.362	.868	2.138	.179

^a^ Controlled for countries’ effects

^b^ The Hosmer and Lemeshow Goodness of Fit. Test statistics were: Chi-square = 4.730; df = 8, p = .786. The C-index was: 0.731; 95% CI = .692-.770; Standard error = .020, Asymptotic sig. < .001. Both tests indicated good fit of the multivariable model.

^c^ OR = Odds ratio

^d^ CI = Confidence Interval

In the univariable models, being male, unemployed, having been hospitalised in the past, not having been diagnosed with a psychotic disorder and at least moderate levels of hostility at baseline were significantly associated with higher likelihood of hostility at three months. In the multivariable model, four predictor variables remained significant: living alone, having been hospitalised in the past, not having a psychotic disorder, and scoring at least moderate levels of hostility at baseline.

At three months, 7.9% (AP = 7.1%) (N = 89) patients with psychotic disorders had moderate or high levels of hostility compared with 11.3% (13.6%) (N = 83) patients with non-psychotic disorders; 11.4% (AP = 10.5%) (N = 128) patients who lived alone had moderate or high levels of hostility compared to 5.9% (AP = 7.1%) (N = 44) of other patients; 10.3% (AP = 10.4%) (N = 138) patients who had been hospitalised had moderate or high hostility compared to 6.3% (AP = 6.4%) (n = 31) of other patients.

## Discussion

### Main results

More than 50% of the involuntarily admitted patients showed at least moderate levels of either suicidality or hostility when they were admitted; 14% of suicidality, 46% of hostility, and 6% of both. For most patients suicidality and hostility reduced over time. Only a small percentage of patients were consistently rated as suicidal or hostile (0.8% and 5.2% respectively).

The general trend of substantial suicidality and hostility reduction was found across countries, despite very different legislations, health care systems and practices of coercive treatment [3,12]. The precise extent of improvement showed some variation among countries, but these differences should be interpreted with much caution as the absolute numbers of patients with suicidality or hostility at follow-ups were rather small in most countries.

Very few patients consistently showed moderate or higher levels of suicidality and hostility throughout the study period. Yet, for some other patients symptoms fluctuated over time. Suicidality and hostility tend to decrease in those patients who have them initially and can occur in others who did not show them when they were admitted.

The prediction of suicidality and hostility after three months showed that–in addition to the baseline levels of the given symptom–being diagnosed with a psychotic disorder and better social support, in form of employment and social contacts, predicted more favorable outcomes. These variables predicted differences that were not only statistically significant but also clinically relevant.

### Strengths and limitations

This is the first large scale study analysing to what extent suicidality and hostility decrease after involuntary hospital admission. The large multicenter sample size provided enough statistical power to detect findings of real clinical significance and showed relatively similar tendencies across countries, suggesting that the findings do not depend on specific features of the setting. Suicidality and hostility were assessed by trained researchers who were independent of the clinical teams and therefore without potential bias for justifying the decision of involuntary admission or for demonstrating positive outcomes of treatment. The researchers used standardised instruments and achieved a good inter-rater reliability. Finally, considering both suicidality and hostility enabled us to analyse indicators of risks to oneself and to others in one study. The two studies had similar design which enabled us to fully take advantages of a pooled analysis approach and test associations of a wide range of patient characteristics with suicidality and hostility.

However, the study also had several limitations:

- Less than 50% of the eligible patients were interviewed at baseline. This figure reflects the challenging nature of recruiting acute involuntary patients into research studies [2,5]. During the InvolvE study data was acquired also for those who did not take part in the interviews which was not possible in the EUNOMIA study. The patients interviewed at baseline in the United Kingdom sample were younger and more likely to be male than those who were not interviewed. There were no other significant differences between these two groups [5]. A selection bias might be possible, although age and gender were not predictors of suicidality or hostility in our study.
- With the exception of the United Kingdom, the national samples were recruited in one to five hospitals only, and it remains unclear to what extent the findings are representative for the given country. Non-representativeness may particularly affect the levels of symptoms, whilst associations between patient characteristics and outcomes can be assumed to be more robust against a potential selection bias [13].
- Not all patients were followed up and a selection bias may have influenced the findings. It has been suggested that patients with high risk levels are more likely to maintain contact with services [14,15], and this might also apply to follow-ups in research studies [16].
- We do not have data on the treatments to which the patients were exposed. Hence, we cannot establish which treatments are more effective in reducing suicidality or hostility.
- Since we included only people receiving an involuntary admission, we cannot exclude that the reduction of suicidality and hostility observed in most patients could simply be due to the natural course of the illness.
- Despite our pooled analysis included the largest studies assessing involuntarily admitted patients, both included studies were carried out in Europe both studies were conducted in Europe, therefore generalisability of the findings to other settings will need to be confirmed.
- Patients were excluded if they had a primary diagnosis of dementia or were admitted due to intoxication. This excludes an important subgroup of potentially suicidal and aggressive patients, further limiting the generalizability of the findings.

### Comparison with literature and interpretation of findings

#### Involuntary hospitalisation and reduction in suicidality and hostility

The reduction of suicidality and hostility after involuntary admission is more evident than the improvement of general symptoms and global functioning of patients. Existing observational studies have suggested only limited improvements of general symptoms and minimal, if any, social gains following involuntary admissions [2,5,7].

There are several possible explanations for these differences:

1. Patients with more or less chronic disorders and a consistently poor social situation may be involuntarily admitted because of fluctuating psychopathological risk indicators rather than because of generally high symptom levels. A mere regression to the mean will then show a reduction of risk levels, but not necessarily a substantial improvement of general symptoms or the social situation.
2. Suicidality and hostility may be particularly alarming for clinicians so that they focus treatment on them and, hence, achieve more substantial improvements on these symptoms than on other outcomes.
3. Hospital wards can provide a regulated and protective environment with supervision through staff and contacts with other patients. This setting might have an especially positive effect on suicidality and hostility [17,18].

#### Predictors of sustained risk

Patients diagnosed with a psychotic disorder were less likely to show suicidality and hostility three months after involuntary admission. This finding held true when the influence of baseline suicidality and hostility levels and other patient characteristics were also considered in the analysis.

The high likelihood of the reduction of suicidality in patients with psychotic disorders (four times higher than for other patients) seem to be inconsistent with other studies which have shown a high risk of suicide in these patients, especially after discharge from hospital [19,20]. In many services, patients with psychotic disorders represent the largest single diagnostic group among involuntary admitted patients. Clinicians are likely to be familiar with treating these patients, and experienced in using the appropriate treatment methods. This might result in a greater suicidality and hostility reduction in patients with psychoses [21,22] than in patients with non-psychotic disorders for whom it can be more difficult to find effective treatment methods in in-patient settings.

Having both suicidality and hostility at the time of involuntary admission did not predict a higher probability of having either suicidality or hostility after three months. This is inconsistent with some previous studies in which hostility was predictive of suicidal behavior. Yet, the previous studies were not conducted in involuntary patients [23,24].

In addition to baseline risk levels and the clinical diagnosis of a non-psychotic disorder, social factors were identified as predictors of suicidality and hostility after three months.

The association between unemployment and suicide risk is well documented in the general population [25,26]. The higher levels of suicidality and hostility following discharge in patients who were unemployed confirms this association and suggest that it may also apply to involuntary patients. Further research may explore whether effective vocational rehabilitation, initiated during or after hospital treatment, can reduce suicidality, in addition to potentially improving other health and social outcomes.

Hostility after three months was more frequent among patients with previous hospitalisations, which may reflect a more persistent course of the illness, and among those living alone. Associations between social isolation and levels of hostility have been shown before [27]. Hostility might both lead to social isolation and be influenced by it. Programs to increase social networks of patients with severe mental illness can aim to break this cycle and may be evaluated as to whether they indeed reduce hostility levels.

In the absence of evidence from randomised controlled trials, the findings of this study provide the best available support to date that involuntary admissions are indeed followed by a reduction of suicidality and hostility. Even if these symptoms fluctuate over time and the identified improvements may be influenced by a regression to the mean, involuntary hospital admission is followed by a substantial and clinically relevant reduction in suicidality and hostility, in particular in patients with psychotic disorders.

The findings may inform ethical debates about the justification of involuntary admissions. One major aspect for ethical decision making in clinical practice is beneficence [28]. The substantial improvement of suicidality and hostility can be seen as an important benefit for patients and suggest the beneficence of involuntary admissions.

Future research should explore the exact mechanisms leading to improvements of suicidality and hostility, and identify which treatments are especially effective in facilitating these improvements. Interventions may aim to foster patients’ social inclusion. Getting patients into regular employment and overcoming their social isolation might have the specific benefit of risk reduction. This may require innovative strategies, e.g. using peer support, befriending schemes, and specific social interventions.

## References

[1] Appelbaum PS. Almost a revolution: an international perspective on the law of involuntary commitment. J Am Acad Psychiatry Law. 1997;25:135–47. 9213286

[2] Kallert TW, Katsakou C, Adamowski T, Dembinskas A, Fiorillo A, Kjellin L, et al Coerced hospital admission and symptom change—a prospective observational multi-centre study. PLoS One. 2011; 6:e28191 10.1371/journal.pone.0028191 22140543 PMC3227658

[3] Salize HJ, Dressing H. Coercion, involuntary treatment and quality of mental health care: is there any link? Curr Opin Psychiatry 2005;18:576–84. 16639122 10.1097/01.yco.0000179501.69053.d3

[4] Katsakou C, Priebe S. Outcomes of involuntary hospital admission-a review. Acta Psychiatr Scand. 2006;114:232–41. 16968360 10.1111/j.1600-0447.2006.00823.x

[5] Priebe S, Katsakou C, Amos T, Leese M, Morriss R, Rose D, et al Patients' views and readmissions 1 year after involuntary hospitalisation. Br J Psychiatry. 2009;194:49–54. 10.1192/bjp.bp.108.052266 19118325

[6] Chodoff P. Involuntary Hospitalization of the Mentally Ill as a Moral Issue. Am J Psychiatry. 1984;141:384–9 6703103 10.1176/ajp.141.3.384

[7] Priebe S, Katsakou C, Yeeles K, Amos T, Morriss R, Wang D, et al Predictors of clinical and social outcomes following involuntary hospital admission: a prospective observational study. Eur Arch Psychiatry Clin Neurosci. 2011;261:377–86. 10.1007/s00406-010-0179-x 21181181

[8] Blettner M, Sauerbrei W, Schlehofer B, Scheuchenpflug T, Friedenreich C. Traditional reviews, meta-analyses and pooled analyses in epidemiology. Int J Epidemiol. 1999;28:1–9. 10195657 10.1093/ije/28.1.1

[9] Lukoff D, Nuechterlein K, Ventura J. Manual for the expanded Brief Psychiatric Rating Scale. Schizophr Bull. 1986;12:594–602.

[10] World Health Organization. The ICD-10 classification of mental and behavioural disorders: clinical descriptions and diagnostic guidelines Geneva: World Health Organization; 1993.

[11] American Psychiatric Association. Diagnostic and statistical manual of mental disorders (4th ed., text rev.). Washington, DC: American Psychiatric Association; 2000.

[12] Fiorillo A, De Rosa C, Del Vecchio V, Jurjanz L, Schnall K, Onchev G et al How to improve clinical practice on involuntary hospital admissions of psychiatric patients: suggestions from the EUNOMIA study. Eur Psychiatry. 2011;26:201–7. 10.1016/j.eurpsy.2010.01.013 20965119

[13] Etter JF, Perneger TV. Snowball sampling by mail: application to a survey of smokers in the general population. Int J Epidemiol. 2000;29:43–8. 10750602 10.1093/ije/29.1.43

[14] Steele MM, Doey T. Suicidal behaviour in children and adolescents. Part 2: treatment and prevention. Can J Psychiatry. 2007;52:35S–45S. 17824351

[15] Zivin K, Pfeiffer PN, McCammon RJ, Kavanagh JS, Walters H, Welsh DE. "No-shows": who fails to follow up with initial behavioral health treatment? Am J Manag Care. 2009;15:105–12. 19284807

[16] Marshall M, Crowther R, Almaraz-Serrano A, Creed F, Sledge W, Kluiter H et al Day hospital versus admission for acute psychiatric disorders. Cochrane Database Syst Rev. 2011;(12):CD004026 10.1002/14651858.CD004026.pub2 12535505

[17] Lipsedge M. Clinical risk management in psychiatry. Qual Health Care. 1995;4:122–128. 10151611 10.1136/qshc.4.2.122 PMC1055300

[18] Janofsky JS. Reducing inpatient suicide risk: using human factors analysis to improve observation practices. J Am Acad Psychiatry Law. 2009;37:15–24. 19297628

[19] Kan CK, Ho TP, Dong JY, Dunn EL. Risk factors for suicide in the immediate post-discharge period. Soc Psychiatry Psychiatr Epidemiol. 2007;42:208–14. 17268761 10.1007/s00127-006-0153-0

[20] Fleischhacker WW, Kane JM, Geier J, Karayal O, Kolluri S, Eng SM, et al Completed and attempted suicides among 18,154 subjects with schizophrenia included in a large simple trial. J Clin Psychiatry. 2014;75:e184–90. 10.4088/JCP.13m08563 24717389

[21] Madsen T, Nordentoft M. Changes in inpatient and postdischarge suicide rates in a nationwide cohort of Danish psychiatric inpatients, 1998–2005. J Clin Psychiatry. 2013;74:e1190–4. 10.4088/JCP.13m08656 24434107

[22] Spivak B, Shabash E, Sheitman B, Weizman A, Mester R. The effects of clozapine versus haloperidol on measures of impulsive aggression and suicidality in chronic schizophrenia patients: an open, nonrandomized, 6-month study. J Clin Psychiatry. 2003;64:755–60. 12934974 10.4088/jcp.v64n0703

[23] Oquendo MA, Galfalvy H, Russo S, Ellis SP, Grunebaum MF, Burke A, Mann JJ. Prospective study of clinical predictors of suicidal acts after a major depressive episode in patients with major depressive disorder or bipolar disorder. Am J Psychiatry. 2004;161:1433–41. 15285970 10.1176/appi.ajp.161.8.1433

[24] Giner L, Jaussent I, Olié E, Béziat S, Guillaume S, Baca-Garcia E, et al Violent and serious suicide attempters: one step closer to suicide? J Clin Psychiatry. 2014;75:e191–7. 10.4088/JCP.13m08524 24717390

[25] Blakely TA, Collings SC, Atkinson J. Unemployment and suicide. Evidence for a causal association? J Epidemiol Community Health. 2003;57:594–600. 12883065 10.1136/jech.57.8.594 PMC1732539

[26] Milner A, Page A, Lamontagne AD. Cause and effect in studies on unemployment, mental health and suicide: a meta-analytic and conceptual review. Psychol Med. 2014;44:909–17. 10.1017/S0033291713001621 23834819

[27] Giacco D, McCabe R, Kallert T, Hansson L, Fiorillo A, Priebe S. Friends and symptom dimensions in patients with psychosis: a pooled analysis. PLoS One. 2012; 7:e50119 10.1371/journal.pone.0050119 23185552 PMC3503760

[28] Beauchamp TL, Childress JF. Principles of Biomedical Ethics, 7th edition New York: Oxford University Press; 2013.

